# Potentials and challenges of an integrative medicine protocol in intensive care: the perspective of intensive care nurses

**DOI:** 10.1186/s12906-026-05423-1

**Published:** 2026-06-04

**Authors:** Ilana Berlowitz, Eliane Timm, Jana Ertl, Ottilia Rohrer, Ursula Wolf

**Affiliations:** 1 Institute of Complementary and Integrative Medicine, University of Bern, Bern, Switzerland; 2 Department of Intensive Care Medicine, Bern University Hospital and University of Bern, Bern, Switzerland

**Keywords:** Intensive care, Integrative medicine, Complementary medicine, Herbal medicine, Critical care, Intermediate care, Nursing, External applications, Topical

## Abstract

**Background:**

Patients admitted to intensive care (IC) settings are exposed to a set of inevitable environmental stressors, such as high noise levels, inadequate lighting conditions, long-term immobilization, and prolonged administration of psychopharmaceuticals, the combination of which is known to be associated with clinical challenges, including poor sleep quality, dysregulation of circadian rhythms, agitation, anxiety, disorientation, or delirium. Due to the great complexity of the IC setting, these conditions are difficult to address with conventional means. The goal of the current study was to assess from the perspective of IC nurses the implementation of an integrative medicine approach based on topically applied herbal medicine substances to attend to some of these challenges. External applications of for instance *Rosmarinus officinalis*, *Oxalis acetosella*, and other herbal substances can have beneficial effects on peripheral circulation, sleep cycles, and digestion; they are considered safe with little drug interactions, hence presenting a valid candidate for the IC context.

**Methods:**

Employing a qualitative research approach, we used a series of focus groups with a sample of *N* = 49 specialized IC nurses working at the IC department of the University Hospital Bern, Switzerland. The qualitative data was analyzed using computer-assisted thematic analysis.

**Results:**

Four main theme clusters, each consisting of various sub-clusters, emerged from the analysis. Overall, participants considered the integrative medicine methods a valuable expansion to their therapeutic toolkit, as they offer additional, readily available, non-invasive, and low risk care options they could apply at their own discretion for their often greatly challenged patients. They described favorable experiences, but also reported barriers, like time constraints, or ineffective operational procedures and offered concrete suggestions on how to improve these.

**Conclusions:**

Taken together, while not assessing clinical effects, this work suggests tailored topical herbal medicines were perceived by IC nurses as feasible and acceptable complementary care options in selected situations.

**Clinical trial number:**

Not applicable.

**Supplementary Information:**

The online version contains supplementary material available at 10.1186/s12906-026-05423-1.

## Background

Intensive care units (ICUs) are designed to attend to acutely unstable patients and involve technologies that can monitor and support failing organ systems [1]. Similarly, intermediate care units (IMCUs; also: high-dependency, step-up, or step-down units) accommodate patients with care needs that exceed the capacities of a general ward but are somewhat below those of ICU patients [2]. Having led to exceptional improvements in patient outcomes over the last 70 years [3], the technologies and interventions performed in ICUs and IMCUs are lifesaving. Nonetheless, the highly technologized and sometimes invasive procedures of intensive care (IC)^1^ ipso facto expose patients to challenge, which may lead to iatrogenic complications [4, 5]. Common stressors are related to inadequate light exposure (lack of nocturnal darkness periods, unnatural daytime light) [6, 7], high noise levels from monitors and procedures like mechanical ventilation [8], long-term immobilization, as well as prolonged administration of psychopharmaceuticals (anxiolytics, sedatives, neuroleptics) [4, 9, 10] and associated problems include poor sleep quality (fragmented and superficial sleep that lacks adequate restorative sleep phases [11]), dysregulation of circadian rhythms (e.g., affecting digestion), agitation, anxiety, depressed mood, disorientation, or delirium [4, 10, 12–16]. Psychological stressors related to invasive procedures and fear of death may further aggravate a sense of vulnerability and anxiety, chronic inner tension, and poor sleep [17–19]. Given the great complexity of the IC context, the multiplicity and inevitability of its stressful influences, the challenging clinical consequences are difficult to address. Research has therefore begun to explore novel approaches in this context [20], for instance experimental IC room design concepts to mitigate the impact of abnormal light exposure [21] or early mobilization to counteract IC-acquired weakness and sequelae [22], yet there continues to be great need for tailored therapeutic approaches that can support patients suffering from these conditions, without disrupting the vital functions performed by ICUs and IMCUs. Non-invasive methods from integrative and complementary medicine, could be a promising avenue in this context [23, 24].

The current article describes a pilot project implementing such methods involving topically applied herbal medicines^2^. External application of herbal medicine substances by means of embrocation, massage techniques, or compresses, are an integral part of many traditional (i.e., Indigenous or ancestral), complementary, and integrative medicine systems [26, 27]. According to the US National Institute of Health NCI Dictionary, integrative medicine is defined as “An approach to medical care that recognizes the benefit of combining conventional (standard) therapies (such as drugs and surgery) with complementary therapies (such as acupuncture and yoga) that have been shown to be safe and effective. […] Integrative medicine tries to address the physical, emotional, social, spiritual, and environmental factors that can affect a person’s health and well-being.” [28]. The approach hence also takes into account the relational component and broader array of factors that may impact a person’s health and well-being [29]. Given their low risk profile, relative simplicity of application, and reported benefits in conjunction with the abovementioned indications, topical application of certain herbal medicines could present a promising approach for the IC context. For example, cutaneous application of rosemary (*Rosmarinus officinalis* L.), a plant used in traditional medicine since earliest times, has been found to stimulate peripheral circulation (particularly its essential oil, see European Medicines Agency’s Committee on Herbal Medicinal Products [EMA/HMPC] [30]). Such effects could be useful in conjunction with the prolonged immobilization of IC patients. Further, the absorption of odorant molecules of rosemary via olfaction [31] is associated with arousing and tonifying effects [32, 33], which might be useful to counteract dysregulated sleep-wake cycles. In a complementary fashion, external application of essential oil derived from lavender (*Lavandula angustifolia* Mill.) is known to aid sleep initiation and restlessness based on their calming and anxiolytic effects [34–37]. Similarly, *Oxalis acetosella* possesses long-standing practical applications in the context of European traditional medicine [38], where leaf extract ointment, placed on the mid or upper abdomen, is employed to support the reestablishment of physiological processes in the digestive system in cases of vegetative dysregulation (be it due to organic or psychological causes, e.g., postoperative or traumatic experience, respectively).

Beyond the specific effects of the herbal substance, which include its effects via local cutaneous absorption and via volatile aromatic constituents (olfaction), external applications also possess therapeutic effects arising from the application method and temperature on vegetative regulation; further, the tactile sensations of embrocation additionally may impart a sense of care. Indeed, a recent multivariate meta-analysis and systematic review demonstrated touch-based interventions’ beneficial effects on physical and mental health variables (e.g., cortisol regulation, reduction of pain, anxiety, depression), some of which could already be shown in newborns [39]. As such, the method has an at least threefold mechanism, with various factors contributing to therapeutic impact [40–42], and is generally are considered safe, with little drug interactions, risks, or contraindications. It hence presents a valid candidate for the IC context, but thus far has been scarcely investigated.

## Methods

### Aims

The overall goal of the current work was to qualitatively assess the implementation process of an integrative medicine protocol involving topical application of herbal medicines to address common clinical complications in IC patients. More specifically, the current study aimed to investigate specialized IC nurses’ subjective experiences and observations regarding feasibility, relevance, significance, and value of these applications after having implemented them in ICU and IMCU wards. 

### Setting: integrative medicine in IC – pilot project

In February 2022 the Institute of Complementary & Integrative Medicine in collaboration with the Intensive Care Unit of the University Hospital Bern, Switzerland, launched a pilot project implementing an integrative medicine protocol aimed to target some common challenges of IC patients. The project was developed in the frame of a close collaboration between clinical and research personnel including both physicians and IC nursing experts (multidisciplinary and participatory approach [43]). The protocol aimed to address some of the previously described indications in adult IC patients, such as sleep disturbances, digestive problems resulting from circadian rhythm disruptions [44], agitation and restlessness, anxiety, tension, fever, or disorientation (see Table 1 for the list of indications), focusing on topically applied herbal medicine substances by means of standardized embrocation, medicinal washes, or compresses, in order to regulate, stimulate, calm, or strengthen different processes [26]. Every application was specifically tailored and adapted to the IC context in terms of indications and contraindications. Most (but not all) of the applications were designed for long-stay patients, for whom the aforementioned conditions are more likely to occur, and often required repeating the application over the course of several days to achieve its full effect. An example would be alternating applications of rosemary in the morning and lavender in the evening over the course of several days to reestablish a normal sleep-wake cycle (for further examples see Table 1). The protocol was designed specifically for implementation by nurses, who after a brief training were authorized to apply the protocol autonomously.

**Table 1:** Indications and associated applications of the protocol

Indication	Herbal medicine substance for external application	Examples of application forms, frequency, and duration
Tiredness, avolition (lack of drive), hypotonia, circulatory weakness	Applications with *Rosmarinus officinalis* (essential oil) as medical body oil (10%) or bath milk	Short embrocation with oil 1–3 times per day; or body wash with water and bath milk once per day; preferably in the morning or during the first half of the day
Restlessness, anxiety	Applications with *Lavandula angustifolia essential oil*) or *Rosa damascene/R. centifolia* (essential oil) as medical body oil (10%) or bath milk	Short embrocation (e.g., feet) with oil 1–3 times per day; or oil cloth (e.g., on chest) for at least 20 min several times per day; or body wash with water and bath milk once per day
Disturbances of sleep-wake rhythm	Applications with *R. officinalis* and *L. angustifolia* as medical body oil (10%) or bath milk	Short embrocation (especially of feet) in the morning (rosemary oil) and evening (lavender oil); body wash with water and bath milk
Sleep disturbances due to anxiety and restlessness	Applications of *L. angustifolia*,* R. damascene/R. centifolia* (essential oil), and *aurum metallicum praeparatum (x4*) as ointment	Ointment cloth over the heart region for at least 20 min, in the evening or during the night
Colic-like pains, spasms	Applications with *L. angustifolia* as medical body oil (10%)	Oil cloth for at least 20 min, several times per day; or, if tolerated, soft embrocation with oil 1–3 times per day
Pain and tensions in the musculoskeletal system, polyneuropathy, tendency to cool limbs, propensity to feel cold	Applications with *R. officinalis* as medical body oil (10%) or bath milk	Short embrocation with oil 1–3 times per day; or oil cloth for at least 20 min, several times per day; or body wash with water and bath milk once per day
Dying process in general or in case of restlessness during the dying process	Applications of *L. angustifolia*,* R. damascene/R. centifolia* (essential oil), and *aurum metallicum praeparatum (x4*) as ointment	Ointment cloth over the heart region for at least 20 min, several times daily
Obstipation or tendency to obstipation (due to organic or other reasons)	Applications of *Oxalis acetosella* as medical ointment (10%)	Ointment cloth for 10–20 min on the mid or upper abdomen (solar plexus region), 1–3 times per day
Treatment after frightening (shocking) event, integration support after an intervention, accompanying measure in case of delirium	Applications of *O. acetosella* as medical ointment (10%)	Ointment cloth for 10–20 min on the mid or upper abdomen (solar plexus region), 1–3 times per day
Fever	Fever wash: 4 l of water with essential oil of *Citrus limon* and *L. angustifolia; C. limon*, *L. angustifolia*, and *Eucalyptus globulus;* or *Citrus limon* alone Or: apple cider vinegar leg compresses (2–3 spoons in 4 l of water)	Fever wash: once or if needed again after 1.5–2 h; Leg compresses: once for 10–20 min and if needed again after 30 min
Nausea	For responsive patients: *Mentha piperita folium* (peppermint) as dried herb infusion; For all patients: applications with *O. acetosella* as medical ointment (10%)	Peppermint infusion to take in sips, hot or cold Oxalis: Ointment cloth for 10–20 min on the mid or upper abdomen (solar plexus region), 1–3 times per day

Except for those for fever, all applications are designed as long-term indication-specific supportive measures and can be used on an as-needed basis when indicated as an adjunctive treatment for up to 4 weeks. General contraindications for external applications of herbal medicines include open wounds or skin lesions in the area of application, as well as allergies to the substances in question. For fever higher than 38° C the applications should not be based on oil but rather use ointment cloths or bath milk in washing water. Some of the herbal substances are not recommended for infants/children or during pregnancy (e.g., rosemary, eucalyptus) and may have further contraindications (e.g., rosemary, eucalyptus use in case of hypertension, epilepsy) or require specific precautions (e.g. lavender use in case of paralytic ileus, bradycardic arrhythmia) and therefore should only be applied by appropriately trained personnel

The pilot project was implemented at the University Hospital Bern’s Intensive Care Department, which consists of both ICU and IMCU wards, has a capacity of 64 patient beds in total, and employs about 220 nurses with varying degrees of training and specialization. The nurse-to-patient ratio in general is estimated to about 1:1.5. The mean age of admitted patients is around 62 ± 16 years and ca. 35% are female. Most admissions (≈ 79%) are not planned. IC patients are treated there for varying timeframes (from 1 to > 20 days, mean length of stay = 2–3 days^3^). Predominant conditions are cardiovascular (≈ 40%), neurological (≈ 19%), respiratory/otolaryngology (≈ 12%), trauma-related (≈ 8%), gastro-intestinal (≈ 8%), and others. Prior to the pilot project’s implementation, a team of integrative medicine specialists (physicians) and IC nursing experts held a series of specialized trainings consisting of in-depth training sessions for each ward’s clinical nursing experts and shorter ones for the rest of the nursing staff. The training provided detailed information on the protocol’s herbal medicine substances, application procedures, indications, and contraindications. It also included practical instructions and hands-on exercises regarding the various types of external applications. A detailed instruction manual and operational guideline as per hospital standard were made available to the nurses and placed on the wards at their disposal.

### Study design

We used a qualitative research approach employing focus groups to explore the various aspects of the implementation process of this integrative medicine protocol from the perspective of IC nurses. The focus group method presents a time-efficient means to generate rich data on the range and diversity of experiences and views held within a target group of individuals or population [45]. Through the co-participation of professionals with insider knowledge, and hence lived expertise that is key to understanding the subject at hand, the focus group method is a useful tool to foster participatory research [46]. The method involves an open form of communication, which harnesses group interactions in favor of in-depth discussions [47] that allow the expression of personal views, in this case the nurses’ experiences and observations regarding feasibility, relevance, significance, value, and meaning of the integrative medicine protocol’s implementation in the ICU and IMCU wards. Since the focus groups were undertaken as a measure of internal quality management at the University Hospital Bern and data analysis took place after full anonymization of the material, the study did not require ethics approval under the applicable Swiss legislation according to the competent authority (*Swissethics*). All participants provided oral informed consent to participate.

### Data collection and sampling

As a first step we developed a list of key questions to guide the focus group discussions (see Table 2) drawing on the relevant literature (e.g., [48–51]) and on discussions in the transdisciplinary work group, which consisted of two experienced physicians who are experts in integrative medicine (research), an IC clinical nurse specialist, and a psychologist experienced in qualitative research. Data collection took place between October and November 2022. Using a convenience sampling approach for the focus groups, we included all nurses working at the IC department of the University Hospital Bern (ICU and IMCU wards) who volunteered to participate in the study. All participants were thoroughly informed about the study via oral and written information and provided oral informed consent in case of participation. Based on the principle of saturation and general recommendations for focus group parameters [52, 53], a total of four groups with a duration of 40–50 min each were conducted. Sample size was initially envisaged based on recommendations from the literature, with around three to six focus groups often described as a likely to generate adequate data and saturation in nursing research, and with a sample number on the lower end usually needed in a context such as ours with homogenous participant groups and a short catalogue of questions to be discussed [54–56]. Subsequently, the degree of saturation was continuously assessed by the investigators who held and transcribed the discussion groups. They observed significant repetition of themes after two to three groups, and after the fourth group, given the lack of substantially novel themes arising (saturation), decided to discontinue data collection. Similarly, in terms of number of participants per group we considered the literature’s recommendations (e.g., of 8–12 [57], or 3–21 [46]) as a general guideline, but then also took into account practical considerations, such as the extent of volunteering participants and their respective availabilities based on their work schedules. The focus group discussions were held in a noise-free room at the IC nursing facilities an hour before the start of the evening shift and were facilitated by an experienced physician (JE), while a psychologist (ET) took observational notes. The facilitator first briefly introduced the topic and then proceeded to moderate the discussion with the help of the previously developed key questions. Rather than using a highly structured frame for managing group dynamics, we adopted a relatively open and non-directive approach, in which participants were free to engage in the discussions to the extent they felt comfortable with, as is generally recommended in a research context such as the one at hand [58]. All focus group discussions were audio recorded.

**Table 2:** Key questions for guiding the focus group discussions

What are your general opinions regarding the program?
Which applications have you used since its introduction, which ones less, and why?
Which conditions need to be met for you to apply the protocol, aside from the indications?
Are there difficulties in the implementation? Which barriers could you identify?
What works well, what is positive?
What could be improved? What would need to change to increase the ease of implementation?
What are your observations on effects on patients?
Who can you contact in case of doubts?

### Data analysis

The recorded discussions were first transcribed verbatim in anonymized form. The material was then coded on the basis of thematic analysis [59] to organize the data set and offer a detailed account of the nurses’ perspectives, using an iterative process. For this purpose, after an initial phase of familiarization with the data, we generated preliminary open codes using *QualCoder* Version 3.2 [60], which were subsequently reduced into more precise thematic clusters to adequately represent the material at hand. While one investigator performed the initial coding, a second investigator reviewed the emerging clusters for thematic accuracy and suggested adaptations to the codes if necessary. A third investigator independently went over the data material to develop their own coding system, which was very similar to the first one. Minor inconsistencies in sub-themes were discussed and agreed upon between the three coders. The adapted coding system was subsequently employed to code the entire data set, performing the final analysis using MAXQDA [61], and the ensuing framework was discussed and agreed upon within the work group. Triangulation was thus achieved by means of investigator triangulation, that is, the implementation of various coders and iterations [62]. In terms of reflexivity, the protocol was designed by UW and OR and implemented by OR and JE. The focus groups were moderated by JE and ET. UW and JE are practicing physicians trained and experienced in integrative medicine, OR is a clinical nurse expert, and ET a research psychologist. The latter performed an initial data analysis, however, to reduce interpretive bias during coding and theme development, a researcher not previously involved in the study (IB) was asked to perform the data analysis independently.

## Results

### Participants

A total of 49 IC nurses took part in this study (an average of *n* = 12 per focus group), of which 28 worked on ICU and 21 on IMCU wards (see Table 3). The large majority (92%) were female. Most participants were IC nurses (49%) or registered IC nurses (39%), and the remainder were IC nurses in training. The participants reported an average of 7.2 years of employment at the University Hospital Bern’s IC Department, which across the sample ranged from 33 years to less than 1 year.

**Table 3:** Participants per focus group discussion

Focus group	*n*	Sex (f/m)	Type of hospital ward	Years of employment at IC Department (m; range)	Nursing education (RICN/ICN/ICN-T)
#1	12	11/1	IMCU	5.5; <1–25	11/1/-
#2	9	8/1	IMCU	7.3;_2–20	8/1/-
#3	16	15/1	ICU	6.2; <1–33	-/12/4
#4	12	11/1	ICU	8.6; <1–22	-/10/2

*N* = 49, *IMCU* intermediate care units, *ICUs* intensive care units, *RICN* registered IC nurse, *ICN* intensive care nurse, *ICN-T* intensive care nurse in training

### Qualitative analysis: main findings

Four main clusters, each consisting of various sub-clusters, emerged from the thematic data analysis (see Table 4), namely *General opinions regarding the integrative medicine in IC pilot project*,* Observations of effects & experiences with the treatments*,* Challenges & barriers for implementation*, and *Optimizing operational procedures.* The following sections describe the content of these main clusters along their sub-themes, using frequent direct quotes to illustrate and exemplify the expressions of participants (labeled FG1-, FG2-, FG3-, or FG4-participants, according to the focus group (FG) in which they participated).

**Table 4:** Main thematic clusters and sub-themes

Main themes and sub-themes	Sample quotes
1. General opinions regarding the integrative medicine in IC pilot project	
1.1 Positive general attitude	*“I consider it a fundamentally good thing if one* […] *combines biomedicine and complementary medicine”*
1.2 Negative general attitude	*“I have to say I’m not such a fan of lavender”*
1.3 Advantages of the integrative medicine protocol	*“I find it great that in cases you’ve tried pharmaceutical medication with no result*,* you can try this route”*
2. Perception of effects & experiences with the treatments	
2.1 Perceived beneficial effects in conjunction with specific herbal applications / specific indications	*“it was a very agitated patient who could not sleep*,* so I gave her the foot massage and then put on an ointment cloth at the end*,* and she was able to sleep a bit afterwards.”*
2.2 No observable effect or uncertain attribution of effects	*“we use not only the complementary medicine protocol*,* but also medications*,* which is why it is difficult to say what has actually had an effect”*
2.3 Most frequent contexts of application	*“patients that are here for seven plus days*,* with anxiety*,* restlessness”*
3. Challenges & barriers for implementation	
3.1 Time constraints	*“when there is time pressure I won’t do it”*
3.2 Not remembering that this option existed	*“it usually just doesn’t occur to me that one could also be doing this”)*
3.3 Sense of insufficient knowledge	“*At the moment I feel insecure about the applications*,* we have lots of neurological patients with head injuries*,* for whom many things are even more delicate”*
3.4 Practical barriers regarding operational processes	“*I have to leave the patient bed and for instance I still need to consult the list to know which application is suitable*,* so during all this time I am not with the patient*”
3.5 Patient may object to the application	“*The barrier could rather also be a patient who does not believe in this method and doesn’t really want to try it”*
4. Optimizing operational procedures	
4.1 Prerequisites needed for successful implementation of the protocol	“*feasible during day shifts mainly*,* as there is more staff; and care work*,* in which it can be integrated*,* is scheduled then*”
4.2 Recommendations for how to improve procedures	“*Perhaps we also need an expertise group for complementary medicine services* […] *who have been trained a bit more extensively perhaps and can support the others on the job”*

#### General opinions regarding the integrative medicine in IC pilot project

Most participants expressed a *positive general attitude* with reference to the pilot project, maintaining that the integrative medicine protocol represented a welcome addition to their available tools which broadened their range of possibilities for attending to the needs of IC patients, as expressed for example by an FG1-participant: “*I find it great that this has been included*,* because we have always also had patient situations where with the regular biomedical measures we have reached a limit*,* so I find the supplementary means fantastic. I’ve already had good feedback or observations*”. Similarly, an FG4-participant stated: *“I consider it a fundamentally good thing if one –if I understand it correctly– combines biomedicine and complementary medicine*”. When *negative general attitudes* regarding the pilot project were expressed, which was not very frequent, they referenced either a general skepticism regarding integrative medicine treatments (e.g., “*I am somewhat lacking the belief in this*”, FG4-participant) or a personal dislike of certain smells (e.g., *“I have to say I’m not such a fan of lavender*,* so when everything smells like lavender*,* I myself end up wanting to leave.“*, FG1-participant).

Many discussions emphasized *advantages of the integrative medicine protocol*, for instance *“I think it gives the nursing staff a bit more scope of action*,* we have more tools we can apply*,* without always requiring a physician straight away.”* (FG1-participant). Aside from broadening the nurses’ scope of action in general, participants also noted that the protocol expanded the variety of care options they could offer to patients, for example *“I find it great that in cases you’ve tried pharmaceutical medication with no result*,* you can try this route; I’ve already had good experiences in this context.”* (FG3-participant). Another speaker pointed out: “*I have the feeling that the people are quite glad when you apply something different*,* something of which they don’t immediately have to be scared – another drug*,* again something they must swallow. There are exceptions*,* of course*.” (FG1- participant). This quote also implies the external applications’ advantage of being low in risk and non-invasive for patients. Further, practicality was mentioned as another advantage of the protocol, as some of the methods require minimal extra time and effort (e.g., “*The ointment cloth is quite practical*,* it takes little time to apply and still generates an effect later*”, FG1-participant) and can be integrated in other nursing tasks (e.g., while performing a washing routine, “*when I wash the legs and then apply a salve*,* I simply add a bit of lavender*,* which in reality is so quickly done*“ FG3-participant). In some discussions the applications’ sensory stimulation was highlighted as an advantage, for example “*In our case usually the neurology patients –and there’s various others who– are not orientated*,* and these applications can be quite useful to activate all the senses*,* I believe. Aside from that*,* I consider the hospital is not such a positive place*,* except for the personnel*,* so it might simply feel good to activate the smell*” (FG1-participant). Olfactory stimulation was also reflected upon by this FG1-participant: *“I can easily imagine that when a patient is lying there for several days and then this very different smell reaches them*,* that it activates the senses and may have some effect”* (see also next section for reports regarding effects). Finally, another participant considered that these methods may have the advantage of facilitating the connection between nurse and patient: “*the patients tend to be stressed*,* they have diagnoses*,* perhaps incurable ones*,* and I don’t know*,* but I believe that if one can find a bit of connection through this and the patient can find some calm*,* or patients that lie for 2–3 weeks here with us*,* yes*,* I believe it is a beautiful option that could be integrated.*” (FG2-participant).

#### Perception of effects & experiences with the treatments

In all the focus groups, *perceived beneficial effects in conjunction with specific herbal applications or specific indications* were discussed. An example was recounted by an FG1-participant: *“I did an application last week during a night shift*,* by means of a foot massage. I don’t know if it was rather the massage or the scent*,* but in any case*,* it was a very agitated patient who could not sleep*,* so I gave her the foot massage and then put on an ointment cloth at the end*,* and she was able to sleep a bit afterwards.”* Another example was described by an FG3-participant: “*Yes*,* for example the fever wash*,* I had the feeling this was quite pleasant for the patient and that it really brought the fever down somewhat*,* when applying it really consistently*,* though.”* An example in which the nurses requested consultation from a physician who is specialized in integrative and complementary medicine for a patient was also discussed: “*We just recently had a patient that was very ill at ease*,* in the sense of agitation. She received a conciliary visit by a complementary*^4^*medicine physician*,* who gave her an ointment on a cloth for the chest*,* which we have then repeated every 8 hours*,* and one could really see* [the effect] *quite impressively in her case; it wasn’t extremely long*,* but for sure for 10–15 minutes she was breathing more calmly and I had the sense one could clearly see an effect.*”(FG4-participant). Some experiences emphasized the olfactory aspect of the medicinal herbs used, pointing out a capacity to ‘transport’ the patients out of the sterile medical environment of the hospital unit, for instance in the words of an FG2-participant: *“I was applying a wash once and then the smell* [of the herbal substance] *went through the room*,* and other patients reacted to it as well. Amid all the technical devices that are present all around at the ward*,* for a moment they were* [transported out], *they said “Ah*,* how lovely”. I had the feeling it just took them out of their monitors and things that are beeping and ringing all the time*,* every day. So it seemed it wasn’t beneficial only for the patient you were applying it on*,* but had a positive effect for everyone around.”* A related experience was reported by an FG4-participant: “*For restless patients I was under the impression that it has shown effects*,* of course not for 8 hours or so*,* but certainly for some time during which relaxation could occur* […] *for instance lavender*,* it may sound silly*,* creates a scent all around the bed which makes everything a bit calmer; I’ve experienced this twice now – I really had the impression that people that came near this bed directly became a bit calmer themselves.”* When participants reported not yet having had the chance to implement the protocol extensively themselves (see also *3.2.3*.), they sometimes recounted second-hand experiences: “*A colleague who uses it regularly described that she can actually observe how individuals can relax better*, [individuals] *that have been rather agitated beforehand*,* or also a reduction in breathing or heart rate*,* which one can observe on the monitor; or that those that have difficulties sleeping are able to sleep for a moment.*” (FG1-participant).

Experiences in which the nurses *observed no effects or were uncertain about the attribution of effects* were also discussed, but with lesser frequency. An example was: “*I have not noticed much success with restless patients*,* although I have a positive attitude; and I find it kind of difficult to check the results or effectiveness*,* because it could also be that it was just me that had the feeling that it didn’t bring the desired success*,* specifically for the restless patients*” (FG4-participant). This quote also alludes to the challenge of determining the effectiveness of an application in conjunction with patients who are not able to provide verbal feedback, which is common in the IC context. Moreover, speakers sometimes expressed that when multiple treatment methods would be applied simultaneously, which in IC patients is often the case, the attribution of any visible effects was uncertain, for example: “*I find it difficult*,* for instance in case of long-term patients* [who are] *agitated*,* we use not only the complementary medicine protocol*,* but also medications*,* which is why it is difficult to say what has actually had an effect*,* was it rather the complementary intervention* [or the medication]. *But in reality*,* this is difficult in general*,* also when we give them several pharmaceutical drugs*,* we cannot know which of them had an effect and which did not”* (FG4-participant). Similarly in this example: “*I once made an abdominal compress with the* [Oxalis] *ointment*,* and naturally we also treated them with a pharmaceutical. The patient was awake*,* it was a neurology patient with a longer course*,* a younger patient*,* and she was subsequently able to have a bowl movement. I feel though that there were several factors that lead to this.”* (FG3-participant). The former expression also recognizes that the integrative medicine protocol was indeed designed to offer means that complement, rather than replace, the available biomedical methods.

With reference to the *most frequent contexts of application*, the nurse practitioners often pointed out patients that lay in the ICU or IMCU for prolonged periods of time (e.g., “*My feeling is that it is needed more by patients that are here for seven plus days*,* with anxiety*,* restlessness*,* or conversely those that are too calm*” FG3-participant), which indeed reflects the reality that the indications, for which the protocol has been designed, are more common in long-term IC patients, as well as that repeated application (which is considered significantly more effective than single uses) are more likely in long-term patients. Some speakers referring to the indications for which they applied the protocol most frequently, such as whole-body washes in case of fever, or foot embrocation with lavender, or ointment cloths (especially if the nurse had little time) in cases of sleep disturbances, restlessness, or anxiety. Or, as FG4-participant put it, *“I’ve also already simply offered an embrocation or a massage when I had the feeling that I want to do something good for the person*,* in the midst of their stressful routine; in cases in which there was room for it and using the corresponding product*.” Finally, another application context that was sometimes mentioned was illustrated by the following case, in which *“*[…] *the relatives wanted to be very present at the patient’s bed and were very concerned about this individual. So*,* a clinical nurse specialist instructed them to do an embrocation*,* something they could offer the patient*,* and they were happy to apply this; it gave them the feeling of ‘at least there’s something I can do’.”* (FG4-participant).

#### Challenges & barriers for implementation

A further point of focus in the discussions included the various challenges and barriers related to the implementation of the protocol. Among these, *time constraints* were prominently discussed in all the groups. The nurses explained that the IC setting is characterized by a chronic sense of time pressure, partially due to the nature of the work, and partially due to understaffing, which leads to instances in which “*I know it would be good* [to use an application from the protocol], *but when there is time pressure I won’t do it to be honest*,* just because I have other things to do.*” (FG3-participant). Similarly in the words of an FG2-participant: “*When it is hectic*,* then this certainly drops away*.” An FG4-participant pointed to further consideration in this context, explaining that sometimes even in cases in which “*I indeed would have the time*,* but the colleague next to me is overloaded with tasks and it would be odd if I go about applying ointments and full-body-washes* […]; *because we rarely don’t have anyone on the ward that is feeling quite bad*,* in which case we help each another out. So*,* I would feel weird if I were to perform these nice applications on my patient*,* while my colleague is in trouble*.” An FG1-participant additionally pointed to IMCU cases in which it is the patient rather than on the nurse that lacks time: *“Especially with the long-term patients there is a lot of interdisciplinary work*,* so all the time physicians enter*,* then physiotherapy*,* then some other professionals*,* and sometimes in the midst of all this stress it seems futile to apply something calming*,* given someone disturbs them all the time anyhow.”*

Furthermore, many speakers noted that, given the protocol was introduced only recently, the applications have not yet become an integral part of their daily routines and were therefore easily forgotten. Thus, an additional barrier was simply *not remembering that this option existed*: *“So it tends to be forgotten*,* at least in my case at this stage*,* and then in retrospect – ‘ah*,* there would have also been this option we could have tried’. It’s not yet very well integrated in our everyday routine”* (FG1-participant). Similar expressions were made by FG4- (“*it usually just doesn’t occur to me that one could also be doing this*”) and FG3-participants (“*At times when it is very hectic*,* an alarm sounding here*,* or you need to go helping there* […] *For me it sometimes gets a bit drowned out by the daily activities to be honest*,* given all the things we do*.”).

Another barrier commonly discussed was a *sense of insufficient knowledge*. Many participants explained that they had missed the training related to the pilot project, be it because of absence on the training days due to part-time employment, or because they only recently started working at the ward (e.g., *“I have also missed the introduction and have only skimmed through the instruction manual”*, FG4-participant). Others considered their recollection of what they had learned in the training was not good enough: “*I would have to fetch the script and read everything to be sure that I indeed apply it correctly*,* I think this is why I don’t use it as often as the patients probably would benefit from it*” (FG3-participant). Some participants maintained that they felt insecure specifically because of potential contraindications (e.g., “*At the moment I feel insecure about the applications*,* we have lots of neurological patients with head injuries*,* for whom many things are even more delicate; then I think I’d rather not do it than do something wrong*”, FG1-participant; or “*respect regarding allergies*”, FG1-participant).

*Practical barriers regarding operational processes* were also frequently discussed in the focus groups. For instance, several participants mentioned that the storage location of the required equipment was inconvenient, which complicated the operational process as well as made it more likely to be forgotten: *„The place where all the material is stored at the moment is simply a cupboard into which one hardly ever looks”* (FG1-participant); *“It is out of the visual field; when we prepare the regular nursing care products*,* it* [material required for applications] *is not in the same place*,* so then you don’t think of it”* (FG1-participant); “*I have to leave the patient bed and for instance I still need to consult the list to know which application is suitable*,* so during all this time I am not with the patient; so I put myself under pressure to do everything quickly*” (FG4-participant). The operational procedures for seeking assistance with the protocol’s treatments were also discussed: “*The clinical nurse specialists are rather far away*,* I mean they are not at the patient bed*,* and when in the short term you* [need help with an application], *you would have to call them –which in the end one often doesn’t*” (FG1-participant). Likewise for an FG2-participant: “*I assume we could ask the clinical nurse specialists*,* but in my view they are not very present*,* in the sense that they don’t tend to just pass by*,* so you could quickly ask them; I feel one would have to actively go and look for them*,* or call them*”.

Finally, only rarely mentioned was that a *patient may object to the application*, which would naturally also represent a barrier: “*The barrier could rather also be a patient who does not believe in this method and doesn’t really want to try it*,* who does not like specific scents.”* (FG2-participant).

#### Optimizing operational procedures

The focus group participants discussed *prerequisites for the successful implementation of the protocol*. A fundamental factor mentioned as a necessary condition was “*the patient’s consent; if the patient objects*,* I cannot apply it*”, (FG1-participant). Further, the importance of the disposal of time or a quiet moment was discussed as necessary for effective implementation: *“Above all time; it makes no sense if I somehow do a massage*,* a foot massage*,* but I do it really rushed –because this then carries over* [to the patient].*”* (FG1-participant). The applications were hence seen as likely more “*feasible during day shifts mainly*,* as there is more staff; and care work*,* in which it can be integrated*,* is scheduled then*” (FG2-participant). Another prerequisite mentioned was related to “*The location* [of the equipment]; *currently it is in the nursing cupboard*,* which is outside the room. Of course it is not kilometers away*,* but still… so the location*.” (FG4-participant). Furthermore, some participants considered the accessibility of experts as an important precondition for successful implementation (e.g., “*probably it just needs a few specialists who are a bit more active*,* individuals who are a bit better trained“*, FG3-participant), and discussed the importance of mutual support as an additional prerequisite: “*I think already just talking about it*,* so it will enter people’s minds*, [reduce] *the fear*,* increase familiarity. Because*,* I have to say*,* with kinesthetics and basal stimulation it was similar in the beginning; when these processes were just recently introduced*,* everyone was scared to apply them too*,* and today they are totally integrated*,* it goes smoothly*” (FG2-participant).

Many participants offered concrete *recommendations for how to improve the operational procedures* to overcome some of the aforementioned barriers. For example, the introduction of a laminated sheet with key information visible at one glance was suggested in all focus groups (e.g., *“I think a laminated sheet on which indications and contraindications are written would be helpful*.“, FG3-participant), expected to greatly support the implementation process: “*In this way one would not have to go looking for the instruction manual*,* especially if it is placed right next to* [where it is needed]” FG1-participant). A visual reminder was considered helpful particularly in these initial phases of the pilot project while the protocol has not yet been fully assimilated by the staff. Some participants further suggested that the integrative medicine treatments should “*perhaps be written into the formal care work plan*,* to be performed at a predetermined time*,* and like this one has it in front of one’s eyes daily*” (FG1-participant). Likewise, an FG3-participant explained: “*If then I can see ‘ah*,* this should be done for this patient every four hours’*,* then a light will flash every four hours*,* and in case I don’t have the time at the indicated moment*,* I can then still postpone it”.* Indeed, an FG4-participant confirmed that *“For the patient that I had mentioned before*,* it was then planned every 8 hours*,* within the care work plan.* […] *like this you actually remember doing it”.* Further, an FG3-participant proposed that the handling process of the material could be simplified in a way that “*products could be premeasured so that one can directly apply them*,* without first having to think; for the handling to get as straightforward as possible*”.

A set of recommendations more related to human resources involved conducting further training activities, such as “*regular refresher courses*” (FG2-participant) to develop and consolidate skills, as well as to reach those that have missed the training in the first instance, such as in this case: *„I have studied the instruction manual a bit more in depth and things are explained really very clearly there*,* but still*,* transmitted by a person it would certainly be even better*,* although the manual is very comprehensible.”* (FG1-participant). A concrete suggestion in this context was to integrate it into the regular continued education curriculum: “*one is regularly summoned to partake in certain continued education courses*,* and if you could also include this topic*,* that would be perfect.”* (FG2-participant). Further, the formation of a *“group of experts in complementary medicine”* (FG4-participant) and the availability of specialists close at the patient bed were also prominently suggested as a means to optimize the implementation process, for instance: “*Perhaps we also need an expertise group for complementary medicine services. Really people who have been trained a bit more extensively perhaps and can support the others on the job”* (FG3-participant). As a reference, some participants pointed to existing IC expertise groups in conjunction with other relevant nursing topics, such as *“basal* [stimulation] *and kinesthetics*,* where we have experts that are present anyway during the day and they approach you; they also know the patients well*,* they work in our teams and also tell the shift manager ‘let me know if you have someone’; or they see a patient lying in bed and say ‘hey*,* this would be a candidate’.”* (FG2-participant). Finally, participants also suggested to foster dialogue on the topic within the team, “*for instance in a team meeting one could perhaps also* [ask] *‘does anyone have a question’*,* see if there is any demand*,* I would find this a good thing*” (FG1-participant), or “*it would be good to have an exchange later*” (FG1-participant).

## Discussion

A number of environmental stressors inherent in IC settings are associated with common but difficult to address clinical consequences, including circadian rhythm and sleep disturbances, agitation, anxiety, disorientation, or delirium [4, 5, 13–16]. The current work reported subjective experiences and observations of IC nurses who were part of a pilot project that implemented an integrative medicine protocol involving topical applications of herbal medicines tailored to some of these challenges of IC patients. We conducted a series of focus group discussions [50] regarding feasibility, relevance, significance, value, and meaning of the integrative medicine protocol’s implementation in the ICU and IMCU wards, using a sample of 49 nurses working at the IC Department of the University Hospital Bern (Switzerland), where the pilot project was being implemented, and subsequently performed thematic analysis [59] of the transcribed qualitative data.

Four main theme clusters, each made of several sub-themes, emerged from the data analysis. In relation to the first, the study revealed a generally positive attitude regarding the integrative medicine protocol among participants. This is in line with international studies showing that IC staff tends to endorse complementary and integrative medicine [63–71]. Some participants also expressed skepticism regarding complementary medicine in general or a personal dislike for the scent of certain essential oils. The nurses perceived the availability of these methods as an advantage when conventional methods had led to little or no relief and valued the increased scope of action. This is significant given IC nurses operate in highly complex environments requiring fast-paced and often critical decisions (around 9 per hour [72, 73]). Indeed, IC nurses’ scope to make discretionary and autonomous decisions has been shown to positively impact their own well-being and be associated with better patient outcomes [74, 75].

The second theme recounted experiences with the protocol and perceptions of effects, including discussions of the most frequent contexts of application (especially patients with long-term IC stays; see [76] for these patients’ care needs), descriptions of clinical cases in which the nurses perceived beneficial effects, no effects, or uncertainty given some IC patients’ limited capacity for expression or since biomedical and herbal treatments are often applied in parallel. Further, some of their descriptions alluded to a multilayered mechanism of action of the herbal applications [40], including perceived favorable effects of the herbal substances via olfaction, in some descriptions not limited to the patient receiving the cutaneous application. The nurses’ perceptions align with a recent systematic review reporting beneficial effects of ‘aromatherapy’ (i.e., absorption of odorant molecules via the olfactory route) on anxiety and sleep quality in IC patients [77]. Earlier studies found inhalation of for instance lavender oil to increase beta power on the electroencephalogram (EEG), associated with sedation and relaxation, as well as diminished contingent negative variation, an indicator for decreased arousal, while rosemary oil on the other hand decreased frontal alpha and beta power, suggesting increased alertness and subjectively stimulating properties [30, 33, 78]. The perceived benefit of physical touch pointed out by some respondents is in line with a systematic review that reported evidence for favorable effects of massage in IC patients, using vital signs, anxiety, and pain indicators as outcomes [79]. Another review investigating various forms of interpersonal touch in IC patients reported significantly lower systolic and diastolic blood pressure and respiratory rate, improved sleep, and decreased pain, concluding that particularly moderate (as opposed to light) pressure touch may elicit a parasympathetic response [80]. Further, as one of the respondents noted, the applications provided an opportunity for non-verbal expression of care, a critical factor for patient well-being [19]. Finally, the impact of direct cutaneous absorption was not reported by the nurses, but would be difficult to percive by mere observation. In the case of lavender, an overview of 30 systematic reviews reported evidence for anxiolytic effects with inhaled, cutaneous (“massage”), as well as oral applications considered promising [37]. Overall, while some nurses reported applications with no perceived effects, no participant reported any adverse effects. Indeed, safety and tolerability of the herbals substance in question have been preestablished via long-standing traditional use [30, 34, 37]; the application method is non-invasive, and can be safely combined with conventional biomedical interventions [40].

The third theme cluster addressed challenges and barriers in the implementation process, among which time constraints due to high workload and understaffing featured prominently. Both of these factors are common in IC settings [81] and present a challenge for the implementation of integrative medicine protocols also in other types of hospital wards [49, 82]. Further, several hindrances seemed to be related to the early stage of the implementation phase, including having missed the training or not remembering it, a general sense of insufficient knowledge, forgetting that the option existed, or practical obstacles embedded in the new operational procedures. Papathanassoglou, Park [83] examined barriers for the utilization of integrative medicine methods in hospital clinicians and, while some of the factors they identified were similar to ours (time constraints, insufficient knowledge), the core barrier to the uptake of these methods in this study was the dominant organizational culture, which created a dissonance between clinicians personal generally favorable views on integrative medicine, versus what they perceived as the organizational view and priorities. Indeed, also more generally, cultural factors may impact nurses’ acceptance of treatments and hence readiness to apply them [82]. Organizational backing, which in our context appears to have been present, may well be an important precondition for successful implementation.

Finally, the fourth theme cluster focused on the optimization of procedures in conjunction with the protocol, identifying prerequisites and concrete suggestions for improvement. In sum, they included creating memory aids with key information, visibility in a strategically opportune locations, refresher training courses to maintain and develop skills, improving ease of access and handling of material, planning the herbal applications as part of the general care work plan, as well as regular presence of specialists on the ward, an internal expert group, and opportunities for exchange on the topic in team meetings. Since these recommendations are broadly applicable, they could be of great value to other healthcare facilities that wish to implement similar protocols.

### Limitations

The current work has various limitations, including its specificity of context and heterogeneity of study population (the pilot project was implemented at a single hospital, thus all participants belonged to the same IC department), which in the absence of comparable data from other facilities and countries might limit the generalizability of our findings. However, given that we investigated general IC wards of a university hospital, and used a sample rather large for qualitative research, we consider that many of the factors identified in the current work may be broadly relevant. Other potential sources of bias may have stemmed from one of the moderators’ involvement in conveying the instructional sessions, thus prior contact with the clinical nursing experts. This had the advantage in-depth expertise and hands-on experience in the subject matter of the moderator, aiding mutual understanding and the elicitation of detailed and relevant information, but as a trade-off may have enhanced social desirability processes, which is however an inevitable potential source of bias, regardless of familiarity with the facilitator. Similarly, bias due to hierarchical differences cannot be ruled out, however, given participants and facilitators had no prior (nor subsequent) work collaboration, were employed in different institutions (University of Bern vs. University Hospital Bern), and geographically distant locations, potential effects of sensed hierarchy may have been limited to those inherent in the relationship of moderated vs. moderating parties common for focus group research. Further, voluntary participation may have preferentially included nurses with greater interest in, or openness toward, integrative approaches, potentially skewing the thematic balance toward favorable views. Clearly, our design does not allow conclusions regarding the effects of the interventions, which was however also not the aim of this study, but which will be essential to obtain through future studies that assess physiological parameters such as heart rate, breathing rate, heart rate variability, digestive activity, and quantitative behavioral ratings (e.g., agitation or restlessness rating scales). Moreover, the current work did not examine patient experiences, which again was not the aim of this study, but which naturally is an essential, but difficult to obtain perspective in the IC context. Future studies should attempt to investigate the patients’ perceived effects via retrospective testimonies, when possible, as well as observations by relatives accompanying the patients in IC. Furthermore, future research could consider an implementation science framework to broaden the exploration of barriers and facilitators [84].

## Conclusions

Overall, this study represents an important contribution to the research literature on integrative medicine methods to address common complications arising from contextual factors in IC patients. We used a multidisciplinary and participatory approach, employing the focus group method to investigate the views of IC nursing staff, that is, of those in charge of the protocol’s implementation, thereby providing a critical perspective for identifying barriers and opportunities for its optimization. While not assessing clinical effects, this work suggests tailored topical herbal medicines were perceived by IC nurses as feasible and acceptable complementary care options for IC patients in selected situations. Overall, the IC nurses expressed appreciation towards these methods as they provided them with additional, non-invasive, and low risk care options, which they can use for their often greatly challenged patients at their own discretion. When such programs are implemented, it is important to take into consideration procedural and organizational barriers, which may be general and somewhat inevitable (e.g., time constraints) or specific to the early stages of implementation (e.g., the need for reminders in daily practice, optimization of operational procedures, or to repeat trainings for new and intermittent staff). Although the current work did not investigate patient outcomes per se, our findings suggest the practicability of external applications of herbal substances in IC settings and their usefulness from the perspective of specialized IC nursing staff.

## Supplementary Information

Supplementary Material 1.

Supplementary Material 2.

## Data Availability

The dataset of the current study will be made available upon reasonable request six months after the publication of study.

## Footnotes

1 We use the term IC inclusively to designate ICUs and IMCUs.

2 The World Health Organization [25] defines *herbal medicines (also: herbal medicine substances)* as preparations, materials, or products made of plant origin or, in some cases, of natural organic or inorganic compounds that are not of plant origin (e.g., mineral or fungal materials).

3 Numerical estimations based on the years 2022–2024.

4 The participants may sometimes use the terms ‘complementary’ and ‘integrative’ medicine interchangeably in their quotes (unlike how the terms were defined at the outset of this paper).

## Abbreviations

IC: Intensive Care
ICUs: Intensive Care Units
IMCUs: Intermediate Care Units
EMA/HMPC: European Medicines Agency’s Committee on Herbal Medicinal Products
