# Supplementary material for: Potentials and challenges of an integrative medicine protocol in intensive care: the perspective of intensive care nurses
Source: BMC Complement Med Ther. 2026 Jun 4;26:238. doi: 10.1186/s12906-026-05423-1 (PMC13449317; doi:10.1186/s12906-026-05423-1)
Supplement: Supplementary file 1 [file 12906_2026_5423_MOESM1_ESM.pdf]

UNIVERSITÄT BERN

Supplementary material 1 - Consolidated criteria for reporting qualitative research (COREQ):

1. Interviewer/facilitator - Which author/s conducted the focus groups? JE and ET.
2. Credentials - What were the researcher's credentials? Dr. med and PhD.
3. Occupation - What was their occupation at the time of the study? Physician and scientific researcher.
4. Gender - Was the researcher male or female? Females.
5. Experience and training - What experience or training did the researcher have? Experience in the treatment methods under investigation, experience in qualitative research, focus groups.
6. Relationship established - Was a relationship established prior to study commencement? All respondents were contacted prior to the focus groups and volunteered to participate,
7. Participant knowledge of the interviewer - What did the participants know about the researcher? That they were a physician and a psychologist working at the Institute of Complementary Medicine of the University of Bern, Switzerland.
8. Interviewer characteristics - What characteristics were reported about the interviewer? That they were an experienced physician (JE) and a psychologist (ET).
9. Methodological orientation and Theory - What methodological orientation was stated to underpin the study? Focus group method; thematic content analysis.
10. Sampling - How were participants selected? Convenience sampling
11. Method of approach - How were participants approached? Via e-mail.
12. Sample size - How many participants were in the study? 49.
13. Non-participation - How many people refused to participate or dropped out? None.
14. Setting of data collection - Where was the data collected? At the respondents' workplace.
15. Presence of non-participants - Was anyone else present besides the participants and researchers? No.
16. Description of sample - What are the important characteristics of the sample? The sample's key characteristics are adequately provided in the text.
17. Interview guide - Were questions, prompts, guides provided by the authors? Yes. Was it pilot tested? Yes.
18. Repeat interviews - Were repeat interviews carried out? No.
19. Audio/visual recording - Did the research use audio or visual recording to collect the data? Yes, audio recording.
20. Field notes - Were field notes made during and/or after the focus groups? Yes.
21. Duration - What was the duration of the focus groups? 40-50 minutes per group, 4 groups (in total 187 min).
22. Data saturation - Was data saturation discussed? Yes, saturation is discussed in the manuscript.
23. Transcripts returned - Were transcripts returned to participants for comment and/or correction? No.
24. Number of data coders - How many data coders coded the data? Three investigators worked on the data analysis (initial coding, reviewing, and adapting).
25. Description of the coding tree - Did authors provide a description of the coding tree? Yes.
26. Derivation of themes - Were themes identified in advance or derived from the data? Preliminary general themes were defined in advance based on the list of key questions, after which themes were derived from the data material itself.
27. Software - What software, if applicable, was used to manage the data? MAXQDA (preliminary analysis with QualCoder).
28. Participant checking - Did participants provide feedback on the findings? No.
29. Quotations presented - Were participant quotations presented to illustrate the themes / findings? Yes. Was each quotation identified? Identification of focus group per quote.
30. Data and findings consistent - Was there consistency between the data presented and the findings? Yes.
31. Clarity of major themes - Were major themes clearly presented in the findings? Yes.
32. Clarity of minor themes - Is there a description of diverse cases or discussion of minor themes? Yes.
