# Supplementary material for: Potentials and challenges of an integrative medicine protocol in intensive care: the perspective of intensive care nurses
Source: BMC Complement Med Ther. 2026 Jun 4;26:238. doi: 10.1186/s12906-026-05423-1 (PMC13449317; doi:10.1186/s12906-026-05423-1)
Supplement: Supplementary file 2 [file 12906_2026_5423_MOESM2_ESM.pdf]

Supplementary material 2

Segment	Initial preliminary coding	Final coding
For instance in a team meeting one could perhaps also [ask] *'does anyone have a question', see if there is any demand, I would find this a good thing*	Recommendations and ideas for improvements	Optimizing operational procedures; Recommendations for how to improve procedures
I am somewhat lacking the belief in this	General opinions – negative opinions	General opinions regarding the integrative medicine in IC pilot project; Negative general attitudes
The place where all the material is stored at the moment is simply a cupboard into which one hardly ever looks	Application – obstacles and preconditions	Challenges & barriers for implementation; Practical barriers regarding operational processes
When it is hectic, then this certainly drops away	Application – obstacles and preconditions	Challenges & barriers for implementation; Time constraints
I find it difficult, for instance in case of long-term patients [who are] *agitated, we use not only the complementary medicine protocol, but also medications, which is why it is difficult to say what has actually had an effect, was it rather the complementary intervention* [or the medication]*. But in reality, this is difficult in general, also when we give them several pharmaceutical drugs, we cannot know which of them had an effect and which did not*	Regarding effects on patients	Perception of effects & experiences with the treatments; No observable effect or uncertain attribution of effects
Yes, for example the fever wash, I had the feeling this was quite pleasant for the patient and that it really brought the fever down somewhat, when applying it really consistently, though	Regarding effects on patients	Perception of effects & experiences with the treatments; Perceived beneficial effects in conjunction with specific herbal applications / specific indications
I find it great that this has been included, because we have always also had patient situations where with the regular biomedical measures we have reached a limit, so I find the supplementary means fantastic. I've already had good feedback or observations	General opinions – positive opinions	General opinions regarding the integrative medicine in IC pilot project; Positive general attitude
The barrier could rather also be a patient who does not believe in this method and doesn't really want to try it, who does not like specific scents	Application – obstacles and preconditions	Challenges & barriers for implementation; Patient may object to the application
